# Magnetotactic bacterial-reprogrammed TCR-macrophages for durable anti-tumor photoimmune therapy

**DOI:** 10.1016/j.mtbio.2026.103512

**Published:** 2026-08-03

**Authors:** Mingze Yan, Ting Yin, Zhiwei Yu, Guojun Huang, Cheng Xiang, Yourong Jiang, Han Gong, Ting Li, Lizhen Zhu, Hongjiang He, Lintao Cai, Hong Pan

**Affiliations:** a Guangdong Key Laboratory of Nanomedicine, CAS-HK Joint Lab of Biomaterials, Guangdong-Hong Kong Joint Laboratory for Metabolic Medicine, Shenzhen Institutes of Advanced Technology (SIAT), Chinese Academy of Sciences, Shenzhen, 518055, China; b Harbin Medical University Cancer Hospital, No. 150 Haping Road, Nangang District, Harbin, Heilongjiang, 150081, China; c Research Center of Nano Technology and Application Engineering, The First Dongguan Affiliated Hospital, School of Pharmacy, Guangdong Medical University, Dongguan, 523808, China

**Keywords:** T cell receptor, Reprogrammed macrophage, Photoimmune response, Tumor microenvironment, Magnetotactic bacteria AMB-1

## Abstract

Macrophage have shown great promise in the clinical treatment for head and neck cancer (HNC). However, macrophage therapies confront significant hurdles due to a deficiency of tumor-specific targets and tumor resistance. Herein, we report the development of a magnetotactic bacterial-reprogrammed T-cell receptor macrophage (TCR-M) that integrates human papillomavirus (HPV) associated HNC specific targeting with innate macrophage infiltration capacity, enabling potent immune cytotoxicity against head and neck squamous cell carcinoma (HNSCC). By internalizing magnetotactic bacteria AMB-1, the TCR-M combination achieves tumor-specific targeting and controllable magnetic responsiveness to elicit durable anti-tumor photoimmune response. Notably, AMB-1 incorporation endows macrophages with magnetic navigation capability and laser-responsive polarization into Bac@TCR-M + L, which acquires M1 phenotypes with sustained anti-tumor photoimmune response. Under magnetic actuation, Bac@TCR-M + L demonstrates significantly enhanced tumor site accumulation compared to untreated TCR-M, accompanied by elevated secretion of TNF-α and IFN-γ and augmented reactive oxygen species (ROS) production. Furthermore, Bac@TCR-M + L re-educates tumor-associated macrophages (TAMs) into anti-tumor M1 phenotypes, achieving collaborative tumor destruction. Our findings establish Bac@TCR-M + L as a paradigm-shifting macrophage therapy that integrates microbial engineering, robotic control, and cell therapy, yielding durable anti-tumor effects in HNC treatment and opening new avenues for overcoming cancer resistance.

## Graphical abstract

**Figure:**
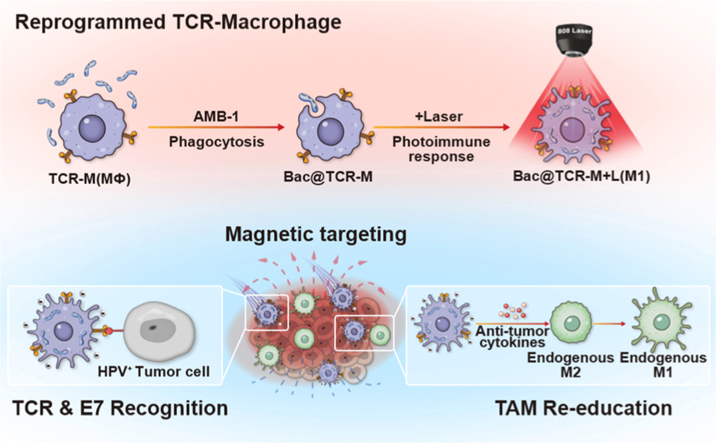

## 1 Introduction

Macrophage-based therapy has exhibited remarkable efficacy in treating head and neck cancer (HNC) [1,2], mainly owing to its unique effector functions—such as exceptional tumor-penetrating capacity and immune effects [[3], [4], [5]]. However, this approach confronts considerable challenges, primarily due to the scarcity of effective cell-recognition targets in malignant HNC. Moreover, immune resistance imposed by the tumor microenvironment (TME) also significantly restricts the therapeutic potency of macrophages [6]. Collectively, these constraints hinder the translational progress of macrophage-based therapeutic strategies for HNC.

Human papillomavirus (HPV) is recognized as a risk factor contributing to the high global incidence of HNC [[7], [8], [9]], and is etiologically linked to the development of head and neck malignancies [10]. HPV evades cell cycle checkpoints via the expression of E6 and E7 oncogenes, which are not expressed in healthy individuals [11]. Researchers have identified a high-affinity T cell receptor (TCR) that specifically recognizes and targets the E7 oncoprotein [8,11,12], positioning it as a promising therapeutic modality for HPV-positive (HPV^+^) HNC in macrophage-based therapy. Meanwhile, other studies have shown that a distinct subset of TCR-expressing macrophages (TCR-M) exhibits markedly enhanced phagocytic activity and secretes abundant cytokines (e.g. TNF-α,IFN-γ, and CCL2) [13,14], thereby mediating potent targeted anti-tumor effects.

On the other hand, the immune resistance caused by the TME also significantly limits the anti-tumor efficacy of macrophage-based therapies for HNC. The TME could reprogram exogenously transplanted macrophages into tumor-associated macrophages (TAMs) that promote tumor progression, diminishing therapeutic efficacy against cancer cells. Remodeling TME and enhancing anti-tumor immunity of exogenous macrophages represents a pivotal challenge. TAMs constitute a substantial immune component and represent a major cellular subset within the TME [4,5,15]. In solid tumors, TAMs typically polarize toward an M2-like phenotype due to microenvironmental cues, including cytokines (e.g., IL-4, CSF1 and VEGF) and metabolic stressors such as lactic acid accumulation and hypoxia [5,16]. This polarization suppresses anti-tumor immunities and facilitates tumor progression. Given TAMs’ pivotal contribution to the establishment of immunosuppressive states within TME, re-educating TAMs to an anti-tumor M1-phenotype to promote macrophage phagocytosis and anti-tumor activities has emerged as a critical therapeutic goal [17,18].

In fact, macrophages possess high phenotypic plasticity and phagocytic capacity, which enables them to respond to stimuli in the tumor environment and switch to anti-tumor phenotype and immune functions [5]. To achieve the above objective, various engineering techniques and stimulatory materials, including metallic, synthetic, and biological agents, have been explored to induce and stabilize M1 polarization of macrophages in the TME, further activating exogenous macrophage anti-tumor immune effects [[18], [19], [20]]. Among these methods, photothermal therapy (PTT) based on biological agents is renowned for its high controllability and non-invasiveness [[21], [22], [23], [24]]. By harnessing the photothermal characteristics of materials, light irradiation triggers a photoimmune response to activate immune activity within the TME and suppress tumor proliferation [[25], [26], [27], [28]]. The photothermal effect induces TAMs polarization toward the M1 phenotype and elicits immunogenic cell death (ICD) in tumor cells [25,29,30]. Concurrently, M1 macrophages exert anti-tumor effects and activate the immune response of the TME by releasing a large amount of anti-tumor-related cytokines [4,25,31]. Based on the above facts, integrating tumor antigen specific TCR with photothermal bio-materials to reprogram macrophages thus represents a promising strategy for the treatment of HPV^+^ HNC.

**Fig. 1:**
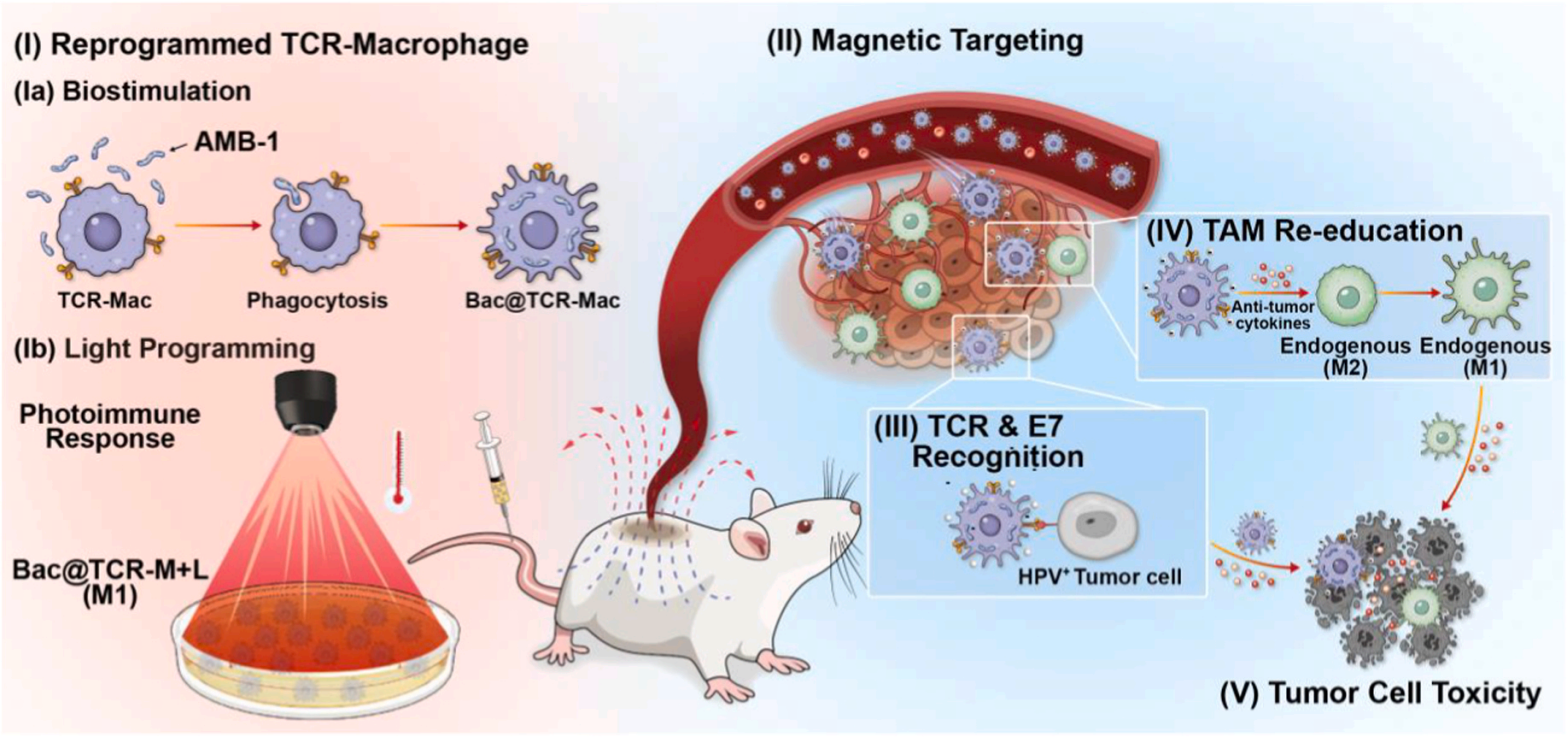
**Scheme outline Magnetotactic bacteria-reprogrammed TCR-macrophages for durable anti-tumor photoimmunity.** (Ia) E7 tumor antigen-targeted TCR-M was generated by lentiviral transferring specific TCR to the macrophages. Subsequently, the TCR-M effectively captured AMB-1 through endocytosis to obtain reprogrammed macrophage (Bac@TCR-M), further polarizing the macrophage into an M1 phenotype under the photoimmune effect of AMB-1. (Ib) Under 808 nm laser irradiation, the resulting micro-heating environment further induced Bac@TCR-M (now denoted Bac@TCR-M + L) to polarize toward anti-tumor M1phenotype. (II) Programmed macrophages precisely targeted and migrated to the tumor site via external magnetic field guidance on internalized AMB-1 within the body. (III) At the tumor sites, reprogrammed macrophages utilized their engineered TCR to specifically recognize E7 antigen and efficiently kill HPV^+^ HNC tumor cells. (IV) Meanwhile, these M1-polarized macrophages secrete anti-tumor cytokines TNF-α and IFN-γ, directly kill tumors and re-educating TAMs. (V) Ultimately, this engineered macrophage strategy achieves synergistic tumor killing and durable immune remodeling of the tumor microenvironment through photoimmune response of reprogrammed macrophages.

In this study, we adopted an innovative approach: lentiviral transduction of TCR, ensuring its stable expression on the surface of TCR-M. Subsequently, AMB-1 bacteria, which possess both magnetic properties and excellent photothermal effects, were phagocytosed by TCR-M cells to form Bac@TCR-M. Laser irradiation was then applied to generate the reprogrammed macrophages (Bac@TCR-M + L). Under the stimulation of AMB-1 and the photothermal effect of laser irradiation, multiple genes and signaling pathways associated with M1-type macrophage polarization were activated [25,32,33]. As a result, Bac@TCR-M + L acquired the M1 phenotype, which is able to activate the photoimmune response and inhibit tumor growth (Fig. 1) [34,35]. Moreover, with the aid of TCR expression and magnetic manipulation, the targeting and killing ability of Bac@TCR-M + L against HPV ^+^ tumor cells was markedly enhanced. Notably, we found that Bac@TCR-M + L can release tumor-killing-related cytokines at the tumor site and maintain a relatively high level within the TME. This not only suppressed tumor growth but also re-educated TAMs [4,5], and specifically polarizing some M2-type macrophages into M1-type macrophages, which exerted a synergistic tumor-killing effect with Bac@TCR-M + L. The reprogrammed macrophage can maintain the M1 phenotype, effectively target and kill tumors, and re-educate TAMs [4,36], thereby activating the immune response within the TME. This magnetotactic bacterial-reprogrammed macrophage represents a novel intelligent cell therapy strategy for the targeted photothermal immunotherapy of HPV^+^ HNC.

## 2 Results and discussion

### 2.1 Preparation and characterization of Bac@TCR-M

**Fig. 2:**
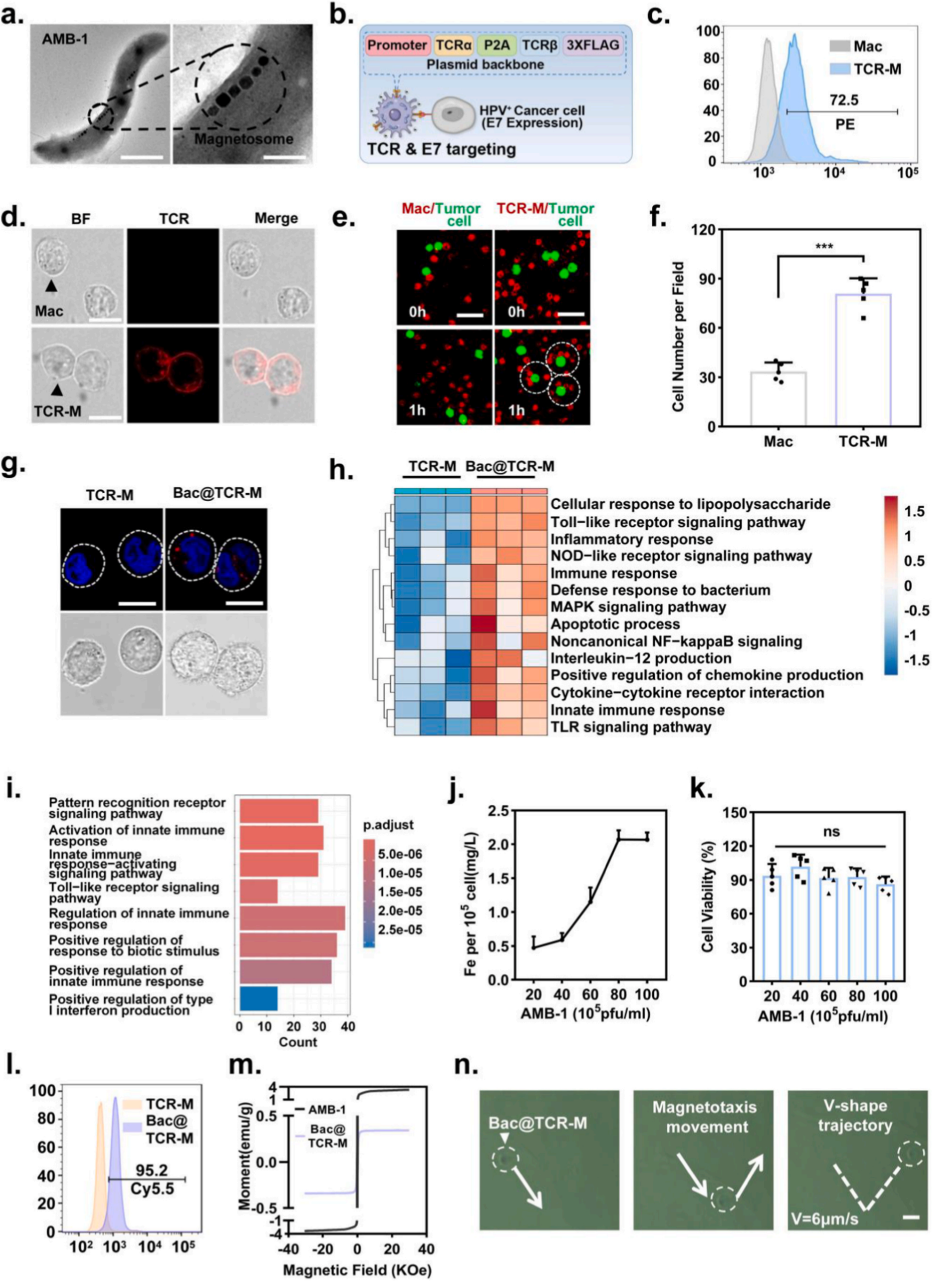
**Preparation and characterization of reprogrammed macrophages.** (a) Transmission electron microscopy (TEM) images of AMB-1 on left (left scale bar = 500 nm) and the magnetosome chains inside AMB-1 on right (right scale bar = 100 nm). (b) Plasmid backbone of designed TCR molecules targeting E7 tumor antigen. (c) Flow cytometry analysis of derived representative image of TCR expression. (d) Confocal imaging analysis of TCR expression on macrophages (left scale bar = 20 μm, right scale bar = 20 μm). (e) Confocal imaging for real-time monitoring of normal macrophage (red) and TCR-M (red) targeting HPV^+^ SCC090 cells (green) (left scale bar = 50 μm, right scale bar = 50 μm). (f) Quantification of macrophages aggregated around tumor cells over time. (g) Confocal microscopy was used to observe the endocytosis of AMB-1 (red) by macrophages (blue) (left scale bar = 20 μm, right scale bar = 20 μm). (h) Heat map of the expression of immune-related genes and the signaling pathway genes. (i) Upregulation of anti-tumor genes related to the stimulation of bacterial in Bac@TCR-M compared with TCR-M by GO analysis. (j) Macrophages were incubated with different concentrations of AMB-1 and detected intracellular iron content by ICP quantitative analysis. (k) Magnetic hysteresis curves of AMB-1(black) and macrophages phagocytizing AMB-1 (blue) at 300K. (l) Influence of macrophage activity under different AMB-1 feeding concentrations. (m) A Bac@TCR-M moved under external variable magnetic field and accurately changed the direction of movement forming a ‘V-shaped ‘trajectory (scale bar = 50 μm). Data are presented as means ± SD. *P < 0.05, **P < 0.01, ***P < 0.001 (n = 3). (For interpretation of the references to colour in this figure legend, the reader is referred to the Web version of this article.)

Natural magnetotactic bacteria exhibit not only active magnetic field-responsive motility but also inherent biological irritability and diverse photothermal properties, offering unique opportunities for the modification of macrophages [[37], [38], [39]]. We first clarified the biological structure of the magnetotactic bacteria AMB-1 via TEM iamging, and clearly observed the distribution of magnetosomes (Fig. 2a). To generate TCR-engineered macrophages (TCR-M), human THP-1-derived macrophages (Mac) were transduced with a lentiviral vector encoding TCRα, a P2A linker, and TCRβ genes (Fig. 2b). Flow cytometry confirmed TCR expression in TCR-M with a 72.5% positive rate (Fig. 2c), further validated by confocal microscopy showing successful TCR (red) expression (Fig. 2d). After transduction, we examined the effect of TCR on macrophage viability to ensure that TCR-M retain favorable cellular activity (Fig. S1). Compared with untreated macrophages, TCR-M showed better targeting effect against HPV^+^ HNC cells (SCC090), and significantly higher cell aggregation per unit area in confocal images (Fig. 2e and f). TCR-M also exhibited excellent phagocytic activity for AMB-1 (red) under co-incubated (Fig. 2g). Time-gradient co-culture assays revealed that AMB-1 exhibits negligible cytotoxicity toward TCR-M under prolonged exposure**.** They can actively phagocytose and internalize AMB-1 to form Bac@TCR-M and progressively degrading them over time (Fig. S2a and S2b). TEM images confirmed the persistence of magnetosomes within Bac@TCR-M (Fig. S3a), which conferred obvious magnetotactic movement under an applied magnetic field (Fig. S3b). Transcriptomic analysis of (heat map and GO enrichment) showed that AMB-1 stimulation significantly upregulated immune responses and gene expression of related signaling pathways in Bac@TCR-M compared to TCR-M alone (Fig. 2h and i; Fig. S4a and 4b). To ensure the rationality and safety of the experiment, we co-incubated TCR-M with AMB-1 bacteria at different concentrations and detected the iron content of Bac@TCR-M at different incubation concentrations by Inductively Coupled Plasma (ICP) analysis. Viability assessment of Bac@TCR-M was evaluated by CCK-8 assay in 24 h to identify 80 × 10^5^ pfu/mL as the optimal AMB-1 concentration (Fig. 2j and k). Meanwhile, we detected the proportion of macrophages that internalized AMB-1under this concentration, and the results showed that over 95% of macrophages successfully internalized AMB-1 (Fig. 2l). Magnetic hysteresis loop results clearly confirmed that Bac@TCR-M inherits the magnetotactic capability of AMB-1 under this concentration (Fig. 2m). Furthermore, Bac@TCR-M demonstrated directional motility under an external magnetic field at a speed of 6 μm/s, dynamically adjusting its trajectory in real-time response to magnetic field changes (Fig. 2n).

### 2.2 Photoimmune effects of reprogrammed macrophages

**Fig. 3:**
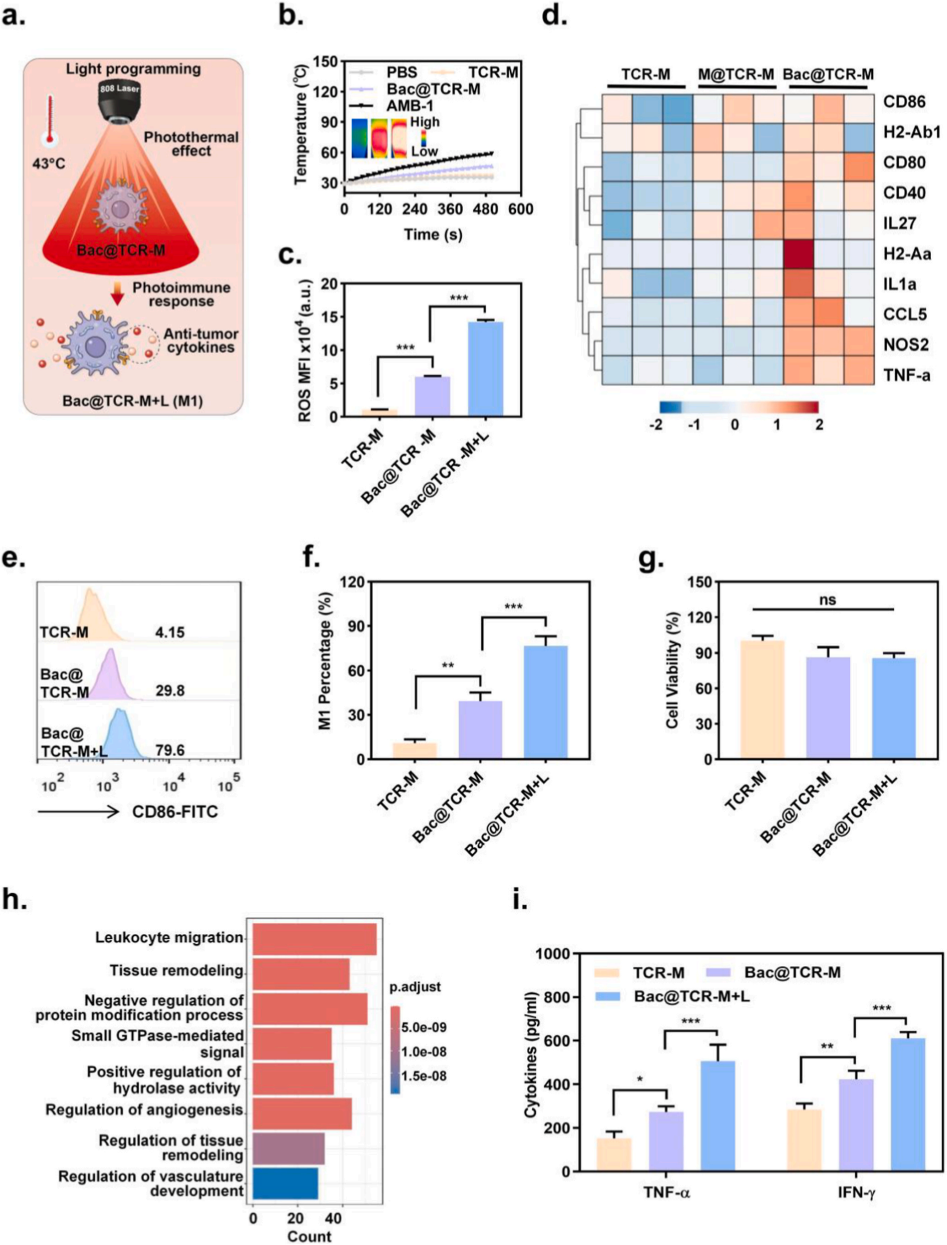
**Photothermal effects of reprogrammed macrophages.** (a) The thermal effects generated by 800 nm laser irradiation induces the immune activation and polarization of Bac@TCR-M towards M1. (b) The temperature change of various substrates occurs under a laser intensity of 0.8 W/cm^2^ over time. (c) Analysis of ROS production levels in each group at 24 h. (d) Heat map of gene expression related to M1-type macrophages in each group. (e-f) Analysis of the M1-type macrophages percentage in TCR-M, Bac@TCR-M and Bac@TCR-M + L. (g) The viability of different types of macrophages were measured to assess the biosafety of bacterial exposure and laser irradiation. (h) Upregulation of genes related to macrophage activity and functionality in Bac@TCR-M + L compared with TCR-M by GO enrichment analysis. (i) Anti-tumor function of various macrophages was assessed by measuring TNF-α and IFN-γ production using ELISA analysis. Data are presented as means ± SD. *P < 0.05, **P < 0.01, ***P < 0.001 (n = 3).

HNC prognosis is negatively correlated with the proportion of M2-type macrophages in TAMs [40,41]. It is crucial to re-educate TAMs into anti-tumor M1-type macrophages [17]. Leveraging the photothermal properties of magnetotactic bacteria, we irradiated Bac@TCR-M with an 808 nm laser at an intensity of 0.8 W/cm^2^ which heated the microenvironment to 43°C. This treatment elevated the environmental temperature and remarkedly activated the anti-tumor functions of Bac@TCR-M, including M1 polarization and subsequent release of various anti-tumor cytokines (Fig. 3a). The temperature changes at various irradiation time-points under indicated laser intensity of 0.8 W/cm^2^ were recorded over time. The Bac@TCR-M group reached 43°C within 360 s and finally 46.7°C, while the other groups did not (Fig. 3b), indicating macrophages internalized magnetotactic bacteria have good photothermal response. Flow cytometry analysis revealed significantly elevated reactive oxygen species (ROS) production in Bac@TCR-M following laser irradiation at 24 h (Fig. 3c). Meanwhile, we found that TCR-macrophages phagocytosing purified magnetosomes (M-TCR-M) also exhibited photothermal effect and ROS generation under identical light conditions, but the response was substantially less pronounced than that of Bac@TCR-M under laser irradiation. This discrepancy may be attributed to the additional biological stimulation provided by intact AMB-1 (Fig. S5a and 5b). Transcriptomic analysis further confirmed that intact AMB-1 more effectively activate photoimmune gene expression related to M1-type macrophages compared to purified magnetosomes alone (Fig. 3d). Furthermore, we also evaluated the percentage of M1-type macrophages in TCR-M, Bac@TCR-M, and laser-treated Bac@TCR-M (Bac@TCR-M + L). Flow cytometry analysis showed that the M1 proportion in Bac@TCR-M + L was approximately 80%, significantly higher than 30% in Bac@TCR-M (Fig. 3e and f; Fig. S6). Importantly, neither bacterial AMB-1 internalization nor laser exposure significantly compromised cell viability (Fig. 3g). GO enrichment analysis revealed that Bac@TCR-M + L significantly upregulated genes associated with macrophage effector function when compared to TCR-M alone, suggesting the photoimmune response of Bac@TCR-M is effectively activated (Fig. 3h; Fig. S7). ELISA detection of anti-tumor cytokines were performed in the macrophages across all indicated groups. The results revealed a significant increase in cytokine production by Bac@TCR-M + L group compared to the Bac@TCR-M control, with 3-fold higher TNF-α and 2.5-fold higher IFN-γ levels (Fig. 3i). These results clearly demonstrated that the synergistic action of internalized AMB-1 and photoimmune response drives the transformation of TCR-M into M1-polarized reprogrammed macrophages with potent anti-tumor activities.

### 2.3 In vitro analysis of anti-tumor activities of reprogrammed macrophages

**Fig. 4:**
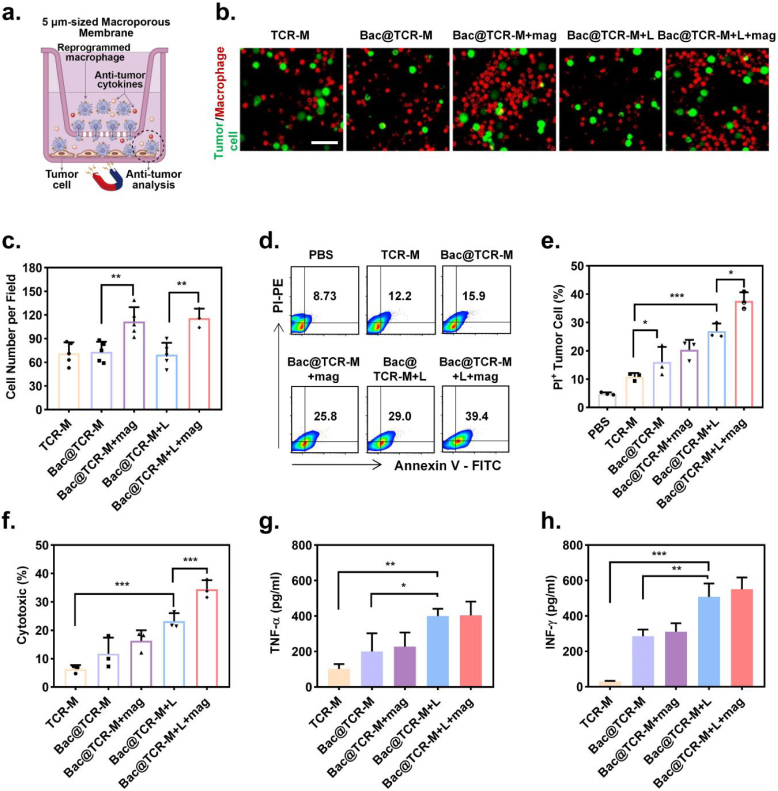
**In vitro analysis of the anti-tumor function of reprogrammed macrophages.** (a) Schematic diagram of the chemotaxis model used for detecting the migration ability of various macrophages (upper chamber) toward HPV^+^ tumor cells (lower chamber) with or without external magnetic field. (b) Confocal microscopy analysis of the aggregation of various macrophages through the microporous membrane to tumor cells (scale bar = 100 μm). (c) Quantification analysis of macrophages with indicated treatment in lower chamber. (d-f) Flow cytometry analysis of tumor cell cytotocicity by various macrophages in the lower chamber. (g-h) The expression levels of TNF-α and IFN-γ were measured by ELISA in the co-culture system containing various types of macrophages. Data are presented as means ± SD. *P < 0.05, **P < 0.01, ***P < 0.001 (n = 3).

To validate the anti-tumor efficacy and immune regulation of reprogrammed macrophages *in vitro*, a transwell model was designed to simulate tumor-targeting and cytotoxic functions of the reprogrammed macrophages. TCR-M, Bac@TCR-M, and Bac@TCR-M + L were separately seeded into the upper chamber of the model, which was separated by a 5-μm-sized microporous membrane. SCC090 cells were added to the lower chamber, and a gradient magnetic field was positioned at the bottom of the chamber to precisely guide the migration of indicated macrophages (Fig. 4a). Following 4 h of treatment, certain macrophages migrated through the microporous membrane into the lower chamber, driven by TCR-mediated targeting and magnetic attraction. Confocal images showed that reprogrammed macrophages exhibited 1.5- to 2-fold higher aggregation around tumor cells per unit area than other groups under the magnetic field condition (Fig. 4b and c). Annexin/PI flow cytometry analysis demonstrated that approximately 20% of lower chamber tumor cells underwent apoptosis following Bac@TCR-M + L treatment. The apoptotic fraction exceeded 30% in the Bac@TCR-M + L group with magnetic field assistance (Bac@TCR-M + L + mag) (Fig. 4d–f). Beyond direct cytotoxicity, we also assessed immune activity via ELISA measurement of anti-tumor cytokines in the supernatant. We found that the Bac@TCR-M + L macrophages produced twice as much TNF-α as Bac@TCR-M cells and 4-fold more than TCR-M cells. Additionally, Bac@TCR-M + L cells secrete 1.5-fold higher IFN-γ than Bac@TCR-M cells and 10-fold more than TCR-M (Fig. 4g and h). The results clearly suggested that photothermal effects induced by laser irradiation significantly improved cytokine secretion of Bac@TCR-M cells.

### 2.4 Reprogrammed macrophages efficiently re-educate TAMs and induce immune activation

**Fig. 5:**
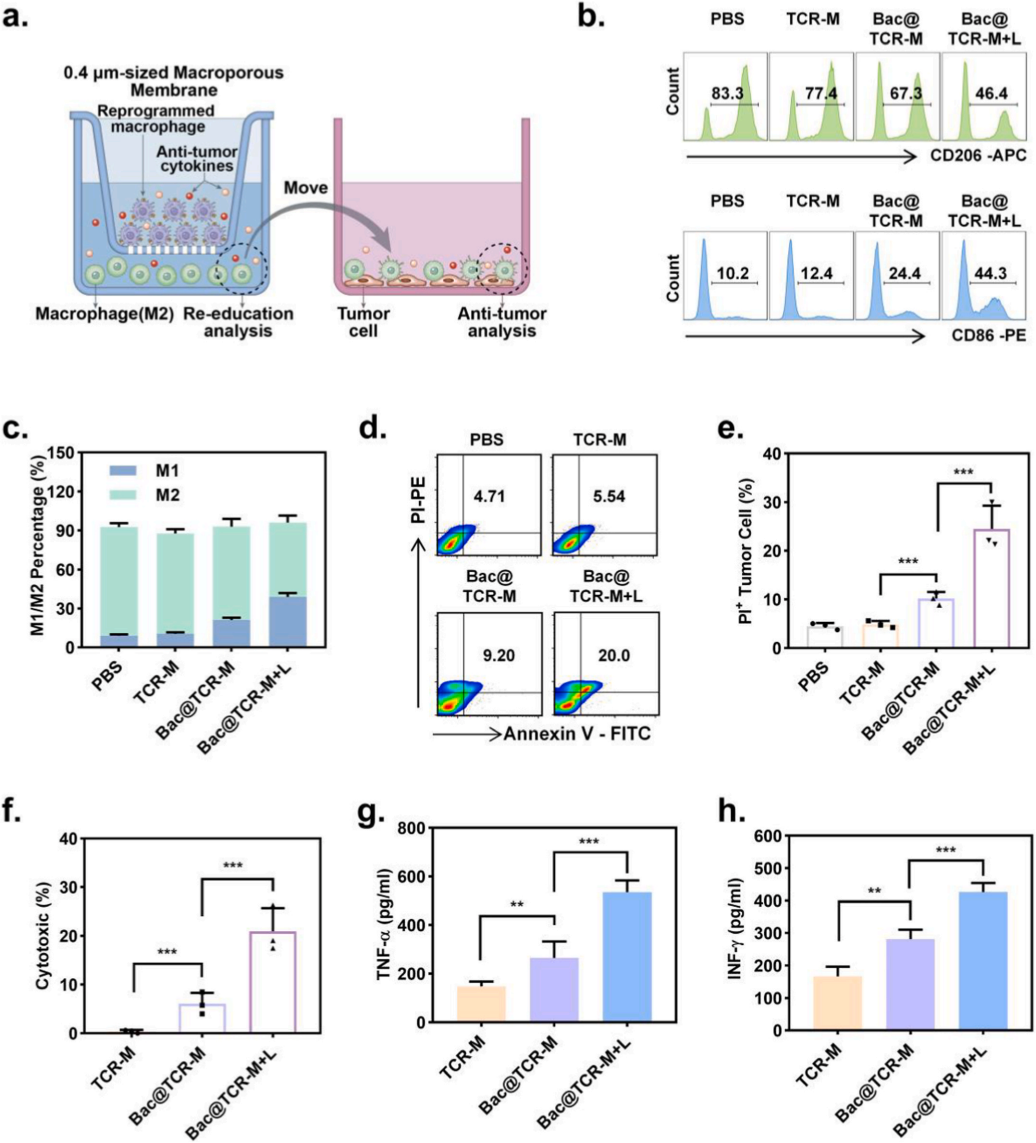
**Reprogrammed macrophages re-educate TAMs and induce immune activation.** (a) Schematic diagram of the re-education ability of various macrophages (upper chamber) toward M2-type macrophages (simulating TAMs) in the lower chamber, as well as the anti-tumor effect of the re-educated M2-type macrophages. (b-c) Flow cytometry analysis of the repolarization proportion of re-educated M2-type macrophages in the lower chamber. (d-f) Flow cytometry analysis of the cytotoxicity effect of re-educated macrophages on tumors in the lower chamber. (g-h) The expression of TNF-α and IFN-γ was dected using ELISA analysis in the co-cultrures with indicated re-educated macrophages. Data are presented as means ± SD. *P < 0.05, **P < 0.01, ***P < 0.001 (n = 3).

Recent studies have reported that photothermal effect plays an important role in the re-education of TAMs and the activation of anti-tumor immunity [4,5,17]. Firstly, to assess the TAMs re-education capability of reprogrammed macrophage, TCR-M, Bac@TCR-M, and Bac@TCR-M + L were separately seeded into the upper chamber of the model, separated from the lower chamber by a 0.4-μm-sized microporous membrane. The lower chamber contained IL-4-polarized M2-type Raw264.7 macrophages, simulating the composition and state of TAMs. Following 24 h of co-culture, the re-educated M2-type macrophages from the lower chamber were collected and reseeded into the upper layer of a new transwell system as depicted in Fig. 4A. HPV^+^ SCC090 cells were placed in the lower chamber as the tumor targets to assess the acquired anti-tumor capability of re-education TAMs (Fig. 5a). Activated M1-type macrophages re-educate TAMs via substantial secretion of anti-tumor cytokines [30]. Flow cytometry analysis of the capacity to re-educate M2-type macrophages within a 24-h co-culture showed that the reprogrammed macrophages can induce polarization of M2-type macrophages towards the M1 phenotype with the Bac@TCR-M + L group showing the most significant effect (∼45% conversion rate) (Fig. 5b and c). Annexin/PI flow cytometry analysis revealed that tumor cells co-cultured with macrophages re-educated by Bac@TCR-M + L exhibited the highest level of apoptosis (∼22%), indicating the most potent anti-tumor effect and re-education capacity (Fig. 5d–f). ELISA measurement results displayed that the concentration of TNF-α and IFN-γ increased 2-3-folds in Bac@TCR-M + L group when compared to other controls, exerting a more significant anti-tumor capability via TAMs re-education (Fig. 5g and h). These data clearly demonstrated that the reprogrammed macrophages sustained a durable photoimmune response, persistently secreted abundant anti-tumor cytokines, and concurrently re-educate M2-type macrophages to polarize into M1 phenotype during tumor cell killing.

### 2.5 In vivo anti-tumor therapeutic efficacy of reprogrammed macrophages

**Fig. 6:**
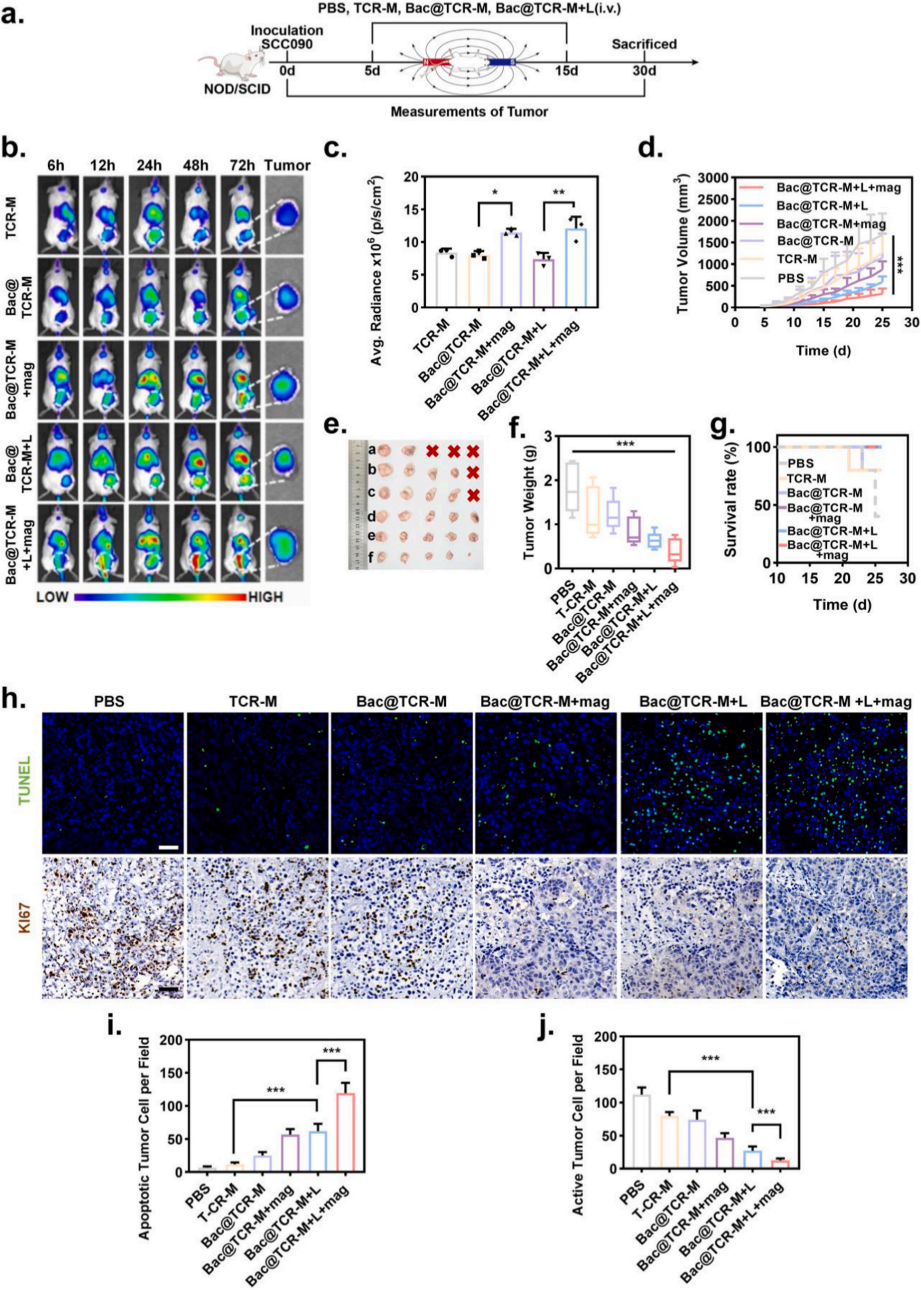
**In vivo anti-tumor effects of reprogrammed macrophages.** (a) The experimental timeline and treatment schema in mouse model. (b) Representing IVIS images depicting biodistribution of various macrophages in vivo with or without external magnetic field over time. The tumor site was designated by white circle. (c) Semi-quantitative analysis of the bio-distribution of various macrophages in NOD/SCID mice based on the average fluorescence intensity of tumor sites in mice. (d) Tumor growth profiles of all groups recorded over time. (e) Representative image of tumor tissues harvested from different groups. (f) Tumor weight of mice in all groups. (g) Survival curves of mice in all groups. (h) Representative immunofluorescence images for TUNEL apoptosis staining (green) (upper scale bar = 200 μm), as well as immunohistochemistry images for Ki67 proliferation staining (brown) (lower scale bar = 200 μm) of tumor sections from different groups. (i-j) Quantitative analysis of the number of apoptotic or proliferative tumor cells within tumor tissue sections within a unit area. Data are presented as means ± SD. *P < 0.05, **P < 0.01, ***P < 0.001 (n = 5). (For interpretation of the references to colour in this figure legend, the reader is referred to the Web version of this article.)

In HPV^+^ SCC090 tumor-bearing mice, macrophages from each group were administrated intravenously via tail vein injection from day 5 to day 15, the experiment was terminated on day 30 post-tumor implantation. For the Bac@TCR-M and Bac@TCR-M + L group, additional subgroups (Bac@TCR-M + mag and Bac@TCR-M + L + mag) received local magnetic field application at the tumor site (Fig. 6a). Each mouse received 5 × 10^6^ DIR-labeled macrophages from each group, and cell biodistribution was intermittently monitored over 72 h using IVIS imaging. Results demonstrated that all groups of macrophages can target tumor tissue by TCR recognition, and the Bac@TCR-M + mag and Bac@TCR-M + L + mag group significantly enhanced in the presence of an external magnetic field (Fig. 6b and c). Another 60-day animal study was carried out to evaluate the off-target effects of long-term treatment with reprogrammed macrophages.The final treatment was performed on day 45, and mice were sacrificed at day 60. IVIS imaging analysis of mouse organs and tumors showed that these cells did not accumulate in normal tissues, suggesting negligible off-target influence on vital organs (Fig. S8). During the 30-day treatment period, tumor size changes and mortality occurrence in each group were recorded at regular intervals. The results indicated that the tumor volume of TCR-M was modest, while both Bac@TCR-M and Bac@TCR-M + L groups progressively inhibited tumor growth. This effect was further augmented in Bac@TCR-M + mag and Bac@TCR-M + L + mag groups, with Bac@TCR-M + L + mag demonstrating the most pronounced tumor-suppression among all groups (Fig. 6d). On day 30, mice were euthanized and tumors were excised for direct comparison of therapeutic outcomes (Group a: PBS, Group b: TCR-M, Group c: Bac@TCR-M, Group d: Bac@TCR-M + mag, Group e: Bac@TCR-M + L and Group f: Bac@TCR-M + L + mag), the tumor volume and weight of the mice treated by Bac@TCR-M + L + mag were minimal compared with other controls (Fig. 6e and f). In contrast to the 80% survival rates of TCR-M and Bac@TCR-M groups, the survival rates of Bac@TCR-M + mag, Bac@TCR-M + L and the Bac@TCR-M + L + mag groups increased significantly to 100% (Fig. 6g). Subsequently, TUNEL (green) and Ki67 (brown) staining were performed on tumor sections from each group, Compared with controls, the Bac@TCR-M + L + mag group exhibited significantly stronger TUNEL staining intensity, accompanied by markedly reduced Ki67 (brown) staining intensity, exhibiting the most potent inhibition of tumor cell growth (Fig. 6h). Quantification of tumor tissues proliferation and apoptosis also confirmed that the anti-tumor efficacy of the Bac@TCR-M + L + mag group was 2-fold that of the Bac@TCR-M + L group and 10-fold that of the TCR-M group (Fig. 6i and j).

During the treatment period, the in vivo biological safety of reprogrammed macrophages was evaluated. Body weight monitoring across all groups revealed no significant fluctuations, indicating the safety of the administrated cell dose (Fig. S9). H&E staining of major organs from each group revealed no histopathological abnormalities, lesions, or degenerative changes in any treatment group (Fig. S10a). And in the 60-day experiment, H&E staining of major organs from therapeutic group also caused no obvious histological damage (Fig. S11a). Concurrently, Serum analysis of hepatic (ALT, AST) and renal (BUN, CRE) function parameters also revealed no significant differences between groups, confirming the absence of notable hepatorenal toxicity (Fig. S10b–8e). Collectively, these results clearly demonstrated the potent in vivo anti-tumor efficacy and favorable biosafety profile of the photoimmune-reprogrammed macrophage therapy.

### 2.6 In vivo TAMs re-education and TME remodeling of reprogrammed macrophages

**Fig. 7:**
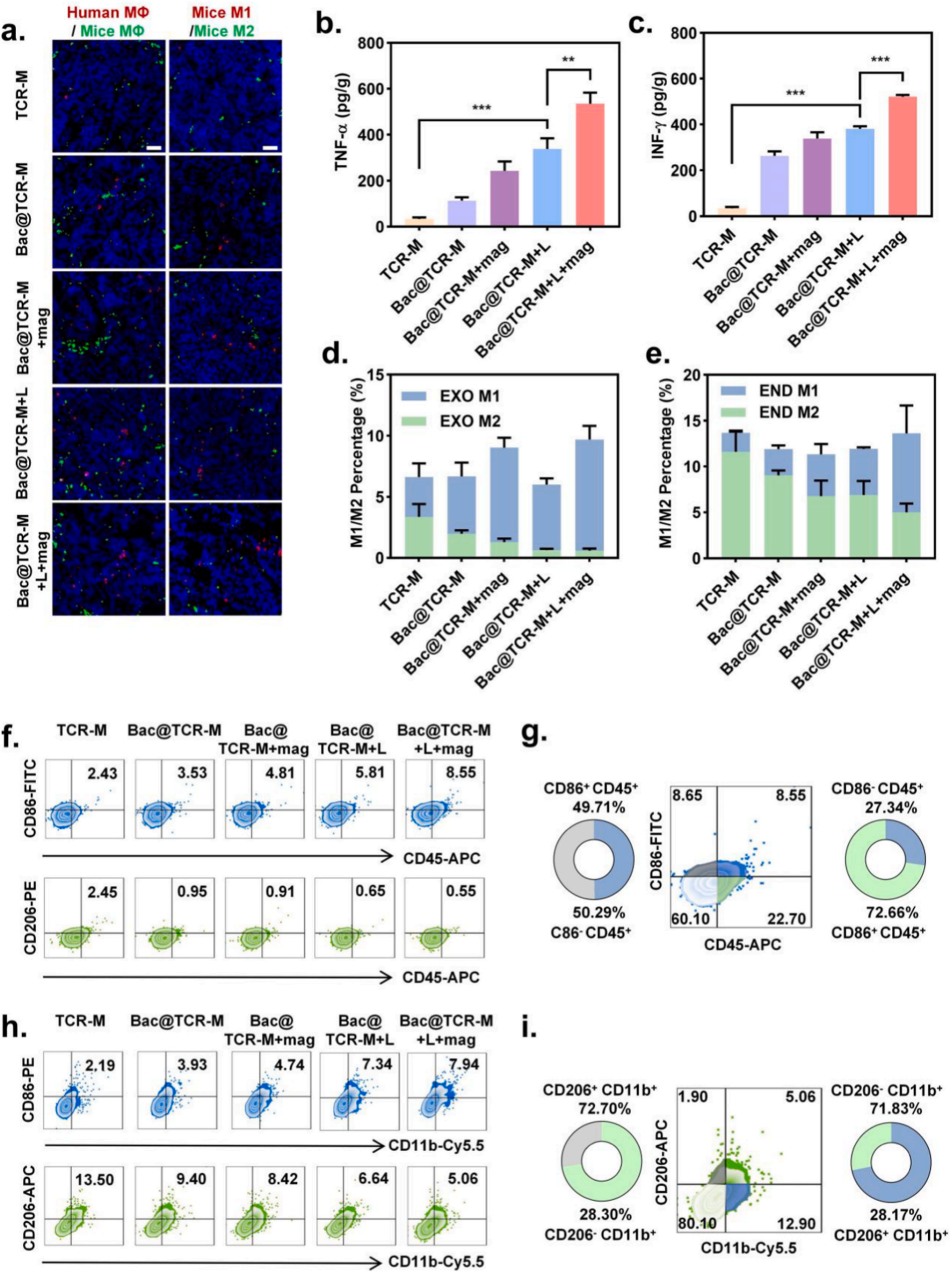
**In vivo re-education of TAMs by reprogrammed macrophages.** (a) Immunofluorescence sections (left column) showed the exogenous macrophages (red) and endogenous macrophages (green) (scale bar = 200 μm). Immunofluorescence sections (righ column) showed the endogenous M1-type macrophages (red) and endogenous M2-type macrophages in TAMs (green) (scale bar = 200 μm). (b-c) The expression levels of TNF-α and IFN-γ in the tumor tissues of mice in each group were measured by ELISA ananlysis. (d) Proportions of M1 and M2 type macrophages from exogenous macrophages in the tumor tissues of mice in each group. (e) Proportions of M1 and M2 type macrophages from endogenous macrophages in the tumor tissues of mice in each group. (f) Representative flow cytometry plots of reprogrammed macrophage in the tumor tissues. (g) Immunophenotype proportion of exogenous macrophages in the tumor tissues of Bac@TCR-M + L + mag group. (h) Representative flow cytometry plots of TAMs in the tumor tissues in each group. (i) Immunophenotype proportion of endogenous macrophages in the tumor tissues of Bac@TCR-M + L + mag group. Data are presented as means ± SD. *P < 0.05, **P < 0.01, ***P < 0.001 (n = 5). (For interpretation of the references to colour in this figure legend, the reader is referred to the Web version of this article.)

Reprogrammed macrophages maintaining M1 phenotype elicited a durable photoimmune response upon tumor homing, further re-educating TAMs and suppressing tumor progression. Immunofluorescence staining was performed on tumor tissue sections of each group to characterize the composition of tumor macrophages. In the left panels, exogenous macrophages were labeled with CD45 (green), while endogenous macrophages were stained with CD11b (red). Results demonstrated that magnetic field application significantly enhanced accumulation of exogenous macrophages at the tumor site in Bac@TCR-M + mag and Bac@TCR-M + L + mag group. In the right panels, endogenous TAMs compositions were assessed by immunostaining with anti-CD86 (red) and anti-CD206 (green), respectively. As shown in Fig. 6A, increased recruitment of M1-type macrophages to the TME effectively promoted progressive endogenous TAMs re-education, as evidenced by a reduced M2-type and an augmented M1-type macrophage population. The Bac@TCR-M + L + mag group exhibited the highest accumulation of exogenous macrophages at tumor sites and the greatest proportion of M1-type macrophages, indicating the most effective TAMs re-education among all groups (Fig. 7a). Meanwhile, the tumor tissues from Bac@TCR-M + L group showed a 10-fold increase in TNF-α and IFN-γ secretion when compared to the TCR-M group, suggesting that reprogrammed macrophages efficiently activate anti-tumor immune responses, and remodel the TME and this immune activation was further enhanced by the targeted actuation of an external magnetic field (Fig. 7b and c). And in the 60-day experiment, no cytokine release syndrome (CRS) was observed in mice, which confirmed that these reprogrammed macrophages provide potent, controllable and safe therapeutic effects (Fig. S11b and 11c). Additionally, flow cytometry analysis further characterized the macrophage composition within tumor tissues of each group. M1-polarized exogenous macrophages in tumor tissues were labeled as CD45^+^/CD86^+^ (blue), while M2-polarized exogenous macrophages were marked with CD45^+^/CD206^+^ (green). Results revealed that the Bac@TCR-M + L + mag group exhibited the highest abundance of M1-type exogenous macrophages at tumor sites, approaching 10% of total cells, more than 3-fold the number of cells detected in the TCR-M group (Fig. 7d and f). Detailed analysis of this group showed that M1-type macrophages constituted over 70% of the reprogrammed macrophages population within the tumor, indicating superior stable immune state and anti-tumor effects of exogenous reprogrammed macrophages (Fig. 7g). Correspondingly, endogenous TAMs composition in each tumor tissue were also characterized. Results clearly displayed that exogenous macrophages promoted TAMs polarization toward the M1 phenotype (CD11b^+^/CD86^+^, blue), most pronounced in the Bac@TCR-M + L + mag group, where approximately 8% of cells were M1-type TAMs, over 3-fold higher than in the TCR-M group (Fig. 7e and h). Further analysis also confirmed that M1-type macrophages constituted over 70% of all endogenous TAMs in Bac@TCR-M + L + mag group, demonstrating the potent re-educating and immunoregulatory effects of reprogrammed macrophages on the TME (Fig. 7i).

## 3 Conclusions

In this study, we reported a novel magnetotactic bacteria-based photoimmune-reprogrammed TCR-macrophage for durable and enhanced antitumor cell therapy. We fabricated Bac@TCR-M + L by internalizing AMB-1 into TCR-macrophages followed by photothermal immune activation. These reprogrammed macrophages achieve precise targeting of HPV^+^ HNC through TCR-mediated E7 antigen recognition and external magnetic field guidance. Crucially, the reprogrammed macrophage Bac@TCR-M maintains M1-type activation and potently activates durable photoimmune responses. While sustaining an activated phenotype, these exogenous reprogrammed macrophages effectively re-educated endogenous M2 TAMs into antitumor M1 macrophages, reversing TME-mediated immune resistance and enabling potent macrophage-mediated antitumor therapy. Building on this artificially reprogrammed macrophage therapy strategy, integrating bioactive materials and photothermal intervention to modulate immune activation represents a highly promising avenue for future macrophage-based therapies.

## 4 Experimental section

### 4.1 Materials

Magnetotactic bacteria (AMB-1) were obtained from Shenzhen Institutes of Advanced Technology (SIAT), Chinese Academy of Sciences. Wolfe's Vitamin Solution and Wolfe's Mineral Solution were purchased from Cosmobiousa LTD. Ferric Quinate, Resazurin, KH_2_PO_4_, NaNO_3_, Ascorbic acid, Tartaric acid, Succinic acid and Sodium acetate were purchased from Aladdin Reagent (Shanghai) Co., LTD. Cell counting kit-8 assay (CCK-8), ROS Brite, and Reactive Oxygen Species Assay Kit were purchased from Beyotime LTD. 1,1-dioctadecyl-3,3,3’,3′- tetramethylindotricarbocyanine iodide (DiR) was purchased from Shanghai BioScience Co. Puromycin was purchased from Coolaber (CHN). PBS, fetal bovine serum (FBS), Penicillin-streptomycin, Trypsin EDTA, RPMI-1640 medium and Dulbecco's Modified Eagle Medium (DMEM) were purchased from Gibco Life Technologies (USA). Recombinant human IL-4, TNF-α ELISA kits and INF-γ. ELISA kits were obtained from Biolegend (USA). APC Cell Viability/Cytotoxicity Detection Kit, TRICT-d-Lys, APC antihuman TCR antibody, APC anti-human CD45 antibody, FITC anti-human CD86 antibody, PE anti-human CD86 antibody, PC5.5 anti-human CD86 antibody, APC anti-human CD206 antibody, PC5.5 anti-mouse CD11b antibody, PE anti-mouse CD86 antibody and APC anti-mouse CD206 antibody were purchased from Biolegend (USA). Transwell plates (0.4 mm and 5 mm pore size) were purchased from Corning.

### 4.2 Cell lines and culture conditions

THP-1 cells were obtained from the American Type Culture Collection (ATCC) (No. YS295C), HPV^+^ SCC090 cells were obtained from ATCC (No. CRL-3240) and RAW 264.7 cells were obtained from ATCC (No. CBP60533). THP-1 cells were cultured in RPMI 1640 medium supplemented with 10% FBS, 1% penicillin-streptomycin, and maintained at 37°C in a 5% CO_2_ incubator. HPV ^+^ SCC090 cells were cultured in DMEM containing 10% FBS, 1% penicillin-streptomycin, incubated at 37°C with 5% CO_2_. TCR-M cells were cultured in RPMI 1640 medium with 10% FBS, 1% penicillin-streptomycin, and 1‰ puromycin (1 g/mL), incubated at 37°C with 5% CO_2_. We confirmed that the cell lines were free of mycoplasma contamination for the described experiments.

### 4.3 Construction of TCR-M

TCR cassettes were cloned into the self-inactivating PGMLV vector using conventional molecular cloning methods. The TCR constructs comprised TCRα chain, P2A peptide linker, TCRβ chain, and EF1α promoter. Lentiviral particles were produced in 293T cells, followed by purification and concentration. All cloning procedures were verified via restriction enzyme digestion and Sanger sequencing. THP-1 cells were transduced with lentivirus at a multiplicity of infection (MOI) of 200 and cultured in RPMI 1640 medium supplemented with 10% FBS, 1% penicillin-streptomycin, and 2 μg/mL puromycin.

### 4.4 Detection of TCR stable expression and targeting function

TCR expression was detected by flow cytometry using an APC-conjugated anti-human TCRβ antibody. For light microscope observation, cells were incubated with the antibody for 30 min before visualization of TCR expression. After stable TCR surface expression was achieved on macrophages, we performed the CCK-8 assay to measure cellular viability at 48 h post-culture, confirming that TCR-M maintain stable cellular viability. Normal macrophages (Mac) and TCR-M were labeled with APC-conjugated anti-human CD45 antibody, while viable SCC090 cells were labeled with PI (propidium iodide) antibody. The labeled Mac and TCR-M were co-incubated with SCC090 cells, respectively, and the targeting efficiency was observed by confocal microscopy (CLSM, TSC SP5II Leica, Ernst-Leitz-Strasse, GER) followed by quantitative cell counting.

### 4.5 AMB-1 culture conditions and purification of magnetosomes

AMB-1 was cultivated in 1.0 L of distilled water supplemented with 10 mL of Wolfe's Vitamin Solution, 5 mL of Wolfe's Mineral Solution, 2 mL of 0.01 M ferric quinate, 0.45 mL of 0.1% resazurin, 0.68 g of KH_2_PO_4_, 0.12 g of NaNO_3_, 0.035 g of ascorbic acid, 0.37 g of tartaric acid, 0.37 g of succinic acid, and 0.05 g of sodium acetate. The pH was adjusted to 6.75 with NaOH, and the culture was incubated at 30°C in a bacterial incubator with shaking at 180 rpm. AMB-1 cells were harvested from the culture medium by centrifugation at 6000–8000 × g for 10-15 min at 4°C. The supernatant was discarded, and the cell pellet was resuspended in ice-cold phosphate-buffered saline (PBS, pH 7.4). The cells were sonicated on ice using a probe sonicator at 300-500 W with 30-s on/off cycles for 5-10 min, using a cooling bath to prevent overheating. The cell lysate was placed in a sterile container, and a strong neodymium magnet (≥1000 G) was applied to the side of the container for 5-10 min. The supernatant (containing debris) was decanted, and the magnetosome pellet was washed 2-3 times with PBS. The magnetosomes were centrifuged at 100,000× g for 1 h to remove any remaining small debris, and the pellet was resuspended in deionized water. The purified magnetosomes were stored in deionized water or PBS at 4°C in a tightly sealed container, away from strong magnetic fields to prevent aggregation.

### 4.6 Construction and characterization of Bac@TCR-M + L

AMB-1 were centrifuged at 5000 rpm for 5 min. The supernatant was discarded, and the pelleted AMB-1 were resuspended in PBS. This centrifugation step (5000 rpm, 5 min) was repeated, with the upper PBS removed each time, followed by resuspension in fresh PBS, repeated three times to obtain purified AMB-1. To determine the optimal bacterial incubation concentration, we co-incubated bacteria with TCR-M at varying concentration gradients for 4 h, generating Bac@TCR-M complexes. The complexes were then washed three or more times with PBS, and cell viability was assessed using CCK-8 assays for each concentration group. Subsequently, samples were prepared for ICP analysis by treating Bac@TCR-M with concentrated nitric acid at a constant temperature of 100°C to digest the samples and quantify the iron content. Based on the results, 80 × 10^5^ pfu/mL was identified as the optimal incubation concentration. We further characterized the magnetic properties of Bac@TCR-M treated at this concentration through hysteresis loop measurements and dynamic cell movement manipulation under variable magnetic fields, with results presented via hysteresis curves and real-time imaging of magnetically driven cell migration. TCR-M was labeled with APC anti-human CD45 antibody, while viable AMB-1 was labeled with TRITC-D-Lys. After 4 h of co-culture, flow cytometry analysis of macrophages was performed, which revealed that over 95% of macrophages internalized AMB-1. The phagocytosis of AMB-1 by TCR-M was visualized via confocal microscopy. A 0–48 h long-term co-culture was performed to test the impact of prolonged exposure on macrophage viability by AMMB-1, and measured cell viability via CCK-8 at all time points. Meanwhile, within 48 h, the distribution of magnetosomes in Bac@TCR-M was detected using TEM (Tecnai G2 20 TWIN transmission electron microscope). To prepare Bac@TCR-M + L, AMB-1 was added at a concentration of 80 × 10^7^ pfu/mL into TCR-M with a concentration of1×10^7^ cells/mL and the concentrations of bacteria and cells were kept consistent across all experiments, incubate for 4 h, centrifuge at 300g for 5 min, discard the supernatant, resuspend with PBS, and repeat washing three times. Suspend the purified Bac@TCR-M in 0.5 mL PBS in a 1.5 mL EP tube, irradiate with an 808 nm laser (0.8W/cm^2^) while oscillating the tube, monitored the temperature with an optical thermometer every time, irradiation was stopped when the temperature reached 43°C to obtain Bac@TCR-M + L, then resuspend in TCR-M medium and culture overnight for further polarization. The ROS intensity of TCR-M, Bac@TCR-M, and Bac@TCR-M + L was detected using ROS Brite at 24 h post-processing. RNA sequencing was performed to analyze and compare gene expression changes related to immune activation, macrophage phagocytic activity, and M1-type macrophage polarization were analyzed and compared among TCR-M, Bac@TCR-M, and Bac@TCR-M + L. These macrophages were stained with FITC anti-human CD86 antibody, and the degree of M1 polarization was detected by flow cytometry every time before use, which confirmed that the functional status of the cells remained stable and met the experimental requirements for treatment. 3 × 10^6^ TCR-M, Bac@TCR-M or Bac@TCR-M + L cells were respectively cultured in 1 mL 1640 medium for 24 h. After centrifugation at 300*g* for 5 min, the supernatant was collected and added to pre-coated 96-well plates. TNF-α and INF-γ ELISA kits were used, and the results were analyzed by a microplate reader.

### 4.7 Assessment of targeting and cytotoxicity of reprogrammed macrophages *in vitro*

1 × 10^5^ SCC090 cells were seeded into the lower chamber of a transwell plate, followed by the addition of 200 μL DMEM medium. Concurrently, 1 × 10^5^ cells each of TCR-M, Bac@TCR-M, and Bac@TCR-M + L were inoculated onto the 5-μm-sized macroporous membrane in the upper chamber. Specifically, the Bac@TCR-M + mag and Bac@TCR-M + L + mag groups were subjected to external magnetic field manipulation. An additional 200 μL DMEM medium was added to each upper chamber. After 24 h of co-culture, the upper chambers were removed. Confocal microscopy was used to observe and count macrophages of each group in unit fields of view within the lower chambers, enabling comparison of the targeting efficiency of cell robots toward tumor cells. The tumor cells in the lower chamber were stained with FITC Annexin V/PE PI and then analyzed by flow cytometry. Take the co-culture medium of each group, after centrifugation at 300*g* for 5 min, the supernatant was collected and added to pre-coated 96-well plates. TNF-α and INF-γ ELISA kits were used, and the results were analyzed by a microplate reader.

### 4.8 Preparation of M2-type macrophages

1 mL of cell suspension (1 × 10^6^ cells/well) was seeded into 24-well plates. Add IL-4 to the cultures at a final concentration of 100 ng/mL. Incubate at 37°C in a 5% CO_2_ atmosphere for 72 h. Replace the medium with fresh IL-4-containing medium every 24 h to maintain cytokine activity. RAW264.7 cells were stained with APC-conjugated anti-mouse CD206 antibody, and the degree of M2 polarization was analyzed by flow cytometry.

### 4.9 Re-education and cytotoxicity of M2-type macrophages co-cultured with reprogrammed macrophages

3 × 10^5^ M2-type macrophages were seeded into the lower chamber of a transwell plate, followed by the addition of 200 μl 1640 medium. Concurrently, 3 × 10^5^ cells each of TCR-M, Bac@TCR-M, and Bac@TCR-M + L were inoculated onto the 0.4-μm-sized macroporous membrane in the upper chamber. Specifically, an additional 200 μl 1640 medium was added to each upper chamber. After 24 h of co-culture, the upper chambers were removed. The part of the various M2-type macrophages were stained with FITC anti-mouse CD86 and APC anti-mouse CD206 antibody, and the proportion of cells re-educated into M1-polarized macrophages was quantified by flow cytometry. Then, M2 type macrophages that have undergone re-education were used to replace the macrophages in the upper chamber, and the tumor-killing experiment described in 4.7 was repeated,and the secretion levels of anti-tumor cytokines TNF-α and IFN-γ in the culture supernatant were detected using ELISA kits.

### 4.10 Establishment of SCC090 tumor bearing mice model

All animal experiments were conducted in accordance with the guidelines of the Animal Care and Use Committee of the SIAT, Chinese Academy of Sciences (Approval No.: SIAT-IACUC-231109-YYS-PH-A2346). Female NOD/SCID mice (6−8 weeks old, obtained from Guangdong Yaokang Biotechnology Co., Ltd., China) were subcutaneously injected with 2 × 10^6^ SCC090 cells per mouse into the right flank region.

### 4.11 Assessment of in vivo biodistribution of reprogrammed macrophages

On day 10 post-tumor implantation, HPV^+^ SCC090 tumor-bearing mice were randomly allocated into 6 groups. Each group was intravenously injected with 5 × 10^6^ DiR-labeled macrophages (TCR-M, Bac@TCR-M, Bac@TCR-M + L or only PBS). Specifically, the Bac@TCR-M + mag and Bac@TCR-M + L + mag groups were subjected to external magnetic field control for 4 h post-injection. The distribution of exogenous macrophages and average fluorescence intensity at tumor sites were monitored using an IVIS imaging system within 72 h after cell administration. And in the 60-day experiment, major organs and tumor tissues were harvested and then examined by IVIS imaging to detect potential off-target signals.

### 4.12 Assessment of anti-tumor effects and photoimmune responses of reprogrammed macrophages

HPV^+^ SCC090 tumor-bearing NOD/SCID mice (6–8 weeks old, female) were randomly divided into 6 groups: PBS, TCR-M, Bac@TCR-M, Bac@TCR-M + mag, Bac@TCR-M + L, and Bac@TCR-M + L + mag. From day 5 to day 15 post-tumor implantation, each mouse received tail vein injections of 5 × 10^6^ macrophages from the corresponding group (150 μL PBS suspension) or 150 μL PBS every 5 days. Mice in the Bac@TCR-M + mag and Bac@TCR-M + L + mag groups were placed in a fixed magnetic field for 4 h after injection. Tumor volume and weight were recorded every other day starting from the first injection. Mice were euthanized at the end of the experiment (day 30 post-tumor implantation) or when reaching IACUC-defined health endpoints, while the 60-day treatment was performed following the same protocols, the final treatment was performed on day 45 and mice were sacrificed at day 60. After in vivo experiments, tumor tissues were ground, digested into single-cell suspensions, and centrifuged (8000 rpm, 10 min) to obtain tumor cells for analyzing exogenous macrophages infiltration and TAM re-education. Cells were stained with anti-human CD45, anti-human CD86, and anti-human CD206 antibodies for flow cytometry analysis of macrophages infiltration. Remaining cells were stained with anti-mouse CD11b, anti-mouse CD86, and anti-mouse CD206 to detect TAM phenotypic shifts. Tumor tissue supernatants were used to assess TNF-α and IFN-γ levels by ELISA kits. Unground tumor sections underwent TUNEL and Ki67 staining to evaluate tumor cytotoxic effects.

### 4.13 Biosafety of reprogrammed macrophages

After the in vivo treatment experiment, peripheral blood was collected and centrifuged (2700 rpm, 30 min) to obtain serum for detecting the levels of liver and kidney functional indices (including AST/ALT and CRE/BUN). Over the 60-day treatment, peripheral blood samples were collected following identical protocols. Several cytokines (including IL-6, IL-1β, TNF-α and IFN-γ) were detected, and no **CRS** was observed. Meanwhile, major organs (heart, liver, spleen, lung, kidney) of each group of mice were harvested for H&E staining to evaluate the biosafety of experimental materials in both 30-day and 60-day experiment.

### 4.14 Statistics analysis

We used GraphPad Prism Version 9.5.1 for statistical data analysis, employing one-way analysis of variance (ANOVA) and t-tests. Results are presented as mean ± standard deviation (SD).

## Ethics approval and consent to participate

All animal experiments were approved by the Institutional Animal Care and Use Committee (IACUC) of Shenzhen Institutes of Advanced Technology (Approval No.:SIAT-IACUC-231109-YYS-PH-A2346).

## CRediT authorship contribution statement

**Mingze Yan:** Conceptualization, Data curation, Formal analysis, Investigation, Methodology, Project administration, Resources, Software, Visualization, Writing – original draft, Writing – review & editing. **Ting Yin:** Conceptualization, Data curation, Formal analysis, Investigation, Methodology, Project administration, Resources, Supervision, Validation, Visualization, Writing – original draft, Writing – review & editing. **Zhiwei Yu:** Conceptualization, Formal analysis, Funding acquisition, Investigation, Project administration, Supervision, Validation. **Guojun Huang:** Conceptualization, Data curation, Formal analysis, Investigation, Methodology, Project administration, Software, Writing – original draft, Writing – review & editing. **Cheng Xiang:** Conceptualization, Data curation, Formal analysis, Investigation, Methodology, Supervision. **Yourong Jiang:** Conceptualization, Data curation, Formal analysis, Investigation, Methodology, Software. **Han Gong:** Conceptualization, Data curation, Investigation, Methodology. **Ting Li:** Data curation, Investigation, Methodology. **Lizhen Zhu:** Conceptualization, Methodology, Software. **Hongjiang He:** Conceptualization, Formal analysis, Funding acquisition, Investigation, Project administration, Supervision, Validation, Visualization. **Lintao Cai:** Conceptualization, Data curation, Formal analysis, Funding acquisition, Investigation, Project administration, Resources, Supervision, Validation, Visualization, Writing – original draft, Writing – review & editing. **Hong Pan:** Conceptualization, Data curation, Formal analysis, Funding acquisition, Investigation, Methodology, Project administration, Resources, Supervision, Validation, Visualization, Writing – original draft, Writing – review & editing.

## Declaration of competing interest

The authors declare no conflict of interest.

## Data Availability

Data will be made available on request.
