# Supplementary material for: Magnetotactic bacterial-reprogrammed TCR-macrophages for durable anti-tumor photoimmune therapy
Source: Mater Today Bio. 2026 Aug 3;40:103512. doi: 10.1016/j.mtbio.2026.103512 (PMC13470447; doi:10.1016/j.mtbio.2026.103512)
Supplement: Supplementary file 1 [file mmc1.doc]

Supporting Information

**Magnetotactic Bacterial-Reprogrammed TCR-Macrophages for Durable Anti-Tumor Photoimmune Therapy**

*Mingze Yan^1, 2^, Ting Yin^3^, Zhiwei Yu^2^, Guojun Huang^1^, Cheng Xiang^2^, Yourong Jiang^1^, Han Gong^1^, Ting Li^2^, Lizhen zhu^1^, Hongjiang He^2*^, Lintao Cai^1*^， Hong Pan^1*^*

**Figure S1.** Cell viability of Mac and TCR-M was measured via the CCK-8 assay.

**Figure S2.** (a) Flow cytometry analyzed macrophage viability post time-gradient incubation. (b) Confocal microscopy revealed that TCR-M (green) phagocytoses AMB-1 (red) when stimulated by Bac@TCR-M and subsequently degrades AMB-1 over time (scale bar =20 mm).

**Figure S3.** (a)TEM image of Bac@TCR-M 24 h later showed black circles labeling free magnetosomes inside (left scale bar =5 μm, right scale bar =500 nm). (b) Bac@TCR-M gradually migrates toward and adheres to the left wall when subjected to a magnetic field.

**Figure S4.** (a-b) The heat map provides a detailed visualization of the significant upregulation of signaling pathways associated with anti-tumor immunity (including TLR and NF-kB pathways, etc.) in Bac@TCR-M upon stimulation with AMB-1.

**Figure S5.** (a) Under the same irradiation intensity of 808-nm laser at 0.8w/cm^2^, the temperature rise of various substrates within the same time, and comperaed their photothermal capabilities. (b) Analyze the levels of ROS production of various reprogrammed macrophages after photothermal action at 24 hours.

**Figure S6.** Compare the levels of M1-type macrophages in the re-induced macrophages of each group, and evaluate the efficacy of magnetosomes and AMB-1 in inducing M1-type polarization of macrophages.

**Figure S7.** RNA sequencing was performed on M@TCR-M+L and Bac@TCR-M+L, and compared the regulation of magnetosomes and AMB-1 on genes related to anti-tumor functional activity in reprogrammed macrophages by GO analysis.

.

**Figure S8.** IVIS imaging analysis of off-target risk on mouse organs and tumors.

**Figure S9.** The body weight changes of each group of mice were recorded and observed during the experiment period.

**Figure S10.** (a) Histological observation of major organs in all groups (scale bar =200 μm). (b-e) Analysis of liver function markers (AST and ALT) and kidney function markers (CRE and BUN) in blood.

**Figure S11.** (a) Histological observation of major organs in therapeutic groups (scale bar =200 μm). (b-c) Analysis of cytokine release syndrome risk (IL‑6, IL‑1β, TNF‑α and IFN-γ）in blood.
